# Correlates of chronic depression in the general population: results from the CoLaus|PsyCoLaus study

**DOI:** 10.1007/s00127-023-02462-8

**Published:** 2023-03-23

**Authors:** Gilles Ambresin, Marie-Pierre F. Strippoli, Caroline L. Vandeleur, Yves de Roten, Jean-Nicolas Despland, Martin Preisig

**Affiliations:** 1 University Hospital of Lausanne, Lausanne, Switzerland; 2 General Practice and Primary Health Care Academic Centre, The University of Melbourne, Melbourne, Australia

**Keywords:** Chronic depression, Depression subtypes, Community study, Cardiovascular risk factors, Personality traits, Coping styles, Adverse life-events

## Abstract

**Purpose:**

Previous population-based studies have partially provided inconsistent results regarding the co-variates of chronic depression, which were likely to be attributable to methodological limitations. The present paper that compared people with chronic major depressive disorder (MDD), non-chronic MDD and no mood disorder in the community focused on specific atypical and melancholic depression symptoms and subtypes of MDD, family history (FH) of mood disorders, measured physical cardio-vascular risk factors (CVRF), personality traits, coping style and adverse life-events.

**Methods:**

Data stemmed from a population-based cohort including 3618 participants (female 53%, n=1918; mean age 50.9 years, s.d. 8.8 years). Among them 563 had a lifetime history of chronic MDD, 1060 of non-chronic MDD and 1995 of no mood disorder. Diagnostic and FH information were elicited through semi-structured interviews, CVRF were assessed through physical investigations.

**Results:**

The major findings were that chronic MDD was associated with increase in appetite/weight and suicidal ideation/attempts during the most severe episode, higher exposure to life-events in adulthood, higher levels of neuroticism, lower levels of extraversion and lower levels of informal help-seeking behavior but less frequent FH of MDD compared to non-chronic MDD.

**Conclusion:**

Chronic MDD is associated with a series of potential modifiable risk factors which are accessible via psychotherapeutic approaches that may improve the course of chronic MDD.

## Introduction

According to population-based research, the duration of at least one out of five depressive episodes exceeds 24 months [1–5], which is usually considered as the threshold for chronicityQuery. Cross-sectional community surveys have established estimates for the lifetime prevalence of chronic depression (CD) ranging from 2.7% in Canada [4], 3.2% in the US (NESARC) [3] to 4.6% in Australia [2]. Applying the criteria for the newly created category of Persistent Depressive Disorders (PDD) of DSM-5 in a random sample from an urban area, we previously reported a lifetime prevalence for PDD with a persistent major depressive episode of 15.2% [6].

Results of recent community studies comparing chronic and non-chronic depression (NCD) suggest that people with CD are older, more often separated, divorced or widowed, more often unemployed and depend on social welfare more often than those with NCD (Table 1) [1–4, 6]. However, results were inconsistent with respect to educational level, income and smoking status (Table 1).

**Table 1:** Socio-demographic and lifestyle characteristics of participants with chronic and non-chronic MDD in community studies

	Satyanarayana 2009	Angst 2009	Rubio 2011	Murphy 2012
*N*	35,323	425	43,093	8841
*n* chronic (lifetime prevalence, %)	1087 (2.7)	55 (5.7)	1439 (3.2)	398 (4.6)
*n* non-chronic (lifetime prevalence,%)	2863 (8.2)	112 (20.9)	4256 (10.1)	967 (10.4)
% chronic among all depressives	27.52	32.93	25.27	29.16
Country	Canada	Switzerland	USA	Australia
Diagnostic assessment	Modified WHO CIDI	SPIKE	AUDADIS-IV	WHO CIDI 3.0
Age-range	> = 15 y.o	19/20–40/41 y.o	> = 18 y.o	18–85 y.o
Chronic depression	Longest MDE > 2 years Any lifetime episode lasting 2 years or more	‘long-term’ major and minor depressive episodes > 2 years, plus work or social impairment	CMDD: MDE > 2 years Lifetime CMDD: a history of > = 1 MDE > 2 years Current CMDD: a history of > = 1 MDE > 2 years, currently symptomatic	Patients with MDD > = 2 years and those with Dysthymic Disorder
Non-chronic depression	Longest MDE < 2 years	2 acute MDE (*N* = 112); 3 minor depressive disorders (*N* = 75); 4 subjects with or without depressive symptoms (*N* = 183)	All other individuals with MDD were classified as having NCMDD	MDD < 2 years
Age (yrs)	–	–	–	C > NC
15–25	C < NC	–	–	–
26–45	C < NC	–	C > NC	–
46–64	C > NC	–	C > NC	–
> = 65	C > NC	–	C > NC	–
Sex (female)	C = NC	C = NC	C = NC	C = NC
Marital status				
Married or common law	C = NC	–	–	–
Widowed, separated, or divorced	C > NC	C = NC	C > NC	–
Single	C < NC	C > NC	C < NC	–
Married or common law	C = NC	–	–	–
Education		C = NC	C < NC	C = NC
< Secondary	C > NC	–	C > NC	-
Secondary graduate	C = NC	–	C > NC	C = NC
> Secondary	C < NC	–		C = NC
Employment				
Housewife	–	C = NC	–	–
Working fulltime	–	C = NC	–	–
Unemployed	C > NC	C = NC	–	C > NC
Housewife	–	C = NC	–	–
Income	–	C = NC	C < NC	–
Disability pension, Social benefits	–	C > NC	–	–
Behavioral characteristics				
Smoking status (current)	–	C = NC	C > NC	–

*C* Chronic, *NC* Non-Chronic, *MDE* Major Depressive Episode, *MDD* Major Depressive Disorder, *CMDD*  Chronic Major Depressive Disorder, *NCMDD* Non-Chronic Major Depressive Disorder, *y.o*  year-old, *yrs*  years, Angst, 2009: Comparison group Episodic (non-chronic) Major Depressive Episode

Conflicting findings were also documented for family history of depression, age of onset and the risk of suicidal attempts (Table 2).

**Table 2:** Family history, course, psychosocial functioning, treatment use, psychological characteristics and life events in participants with chronic and non-chronic Major Depressive Disorder in community studies

	Satyanarayana, 2009	Angst 2009	Rubio 2011	Murphy 2012
Family history				
Depression		C = NC	C > NC	C = NC
Anxiety		C = NC		
Alcohol problems			C > NC	
Drug problems			C > NC	
Behavioral problems			C > NC	
Course				
Age of onset	C > NC	C = NC	C > NC	C < NC
Recurrent episodes	–	–	C > NC	C > NC
Duration most severe episode	–	–	–	–
Number of symptoms most severe episode	–	–	C > NC	–
Duration longest episode	–	–	C > NC	–
Suicide attempts	C > NC	–	–	C = NC
Psychosocial functioning	C < NC	–	–	
Comorbid psychiatric disorders	C > NC	–	C > NC	C > NC
Dysthymia	–	–	C > NC	–
Any anxiety disorder^3^	–	–	C > NC	–
Generalised anxiety disorder	–	C = NC	C > NC	C > NC
Panic disorder	C > NC	C = NC	C > NC	C = NC
Agoraphobia	C > NC	C = NC	–	C = NC
Social phobia	C > NC	C > NC	C > NC	C = NC
PTSD	–	–	–	C > NC
Obsessive–compulsive disorder	–	C = NC	–	C > NC
Substance dependence	C > NC			
Alcohol abuse or dependence	–	C = NC	C = NC	
Illicit substance abuse or dependence	–	C = NC	C = NC (abuse) C > NC (dep.)	C = NC
Psychological characteristics	–	–	–	–
Coping (Mastery)	–	–	–	–
Age 20	–	C < NC	–	–
Age 41	–	C < NC	–	–
Self-esteem	–	–	C < NC	–
Age 20	–	C = NC	–	–
Age 41	–	C < NC	–	–
Life-events	–	–	–	–
Childhood risk factors (parental loss, vulnerable family environment)	–		C = NC	–
Traumatic load	–	–	–	C > NC^1^
Current psychological distress				C > NC^1^
Death of someone close at 1st episode				C > NC^2^
Somatic comorbidities	C > NC	C > NC	–	C = NC^2^
Treatment	–	–	–	C < NC
Outpatient treatment	C = NC	C > NC	C > NC	–
GP/Internist	–	C > NC	–	–
Psychiatrist	–	C > NC	–	–
Psychologist	–	C = NC	–	–
No professional treatment	–	C < NC	–	–
*Any medication*	–	C = NC	C > NC	–
Antidepressants	–	C > NC	–	–
Antipsychotics	–	C > NC	–	–
Mood stabilizers	–	C = NC	–	–
Sedatives	–	C = NC	–	–
*Any hospitalisation*	C > NC	-	C > NC	–

*C* Chronic, *NC* Non-Chronic, *PTSD * post-traumatic stress disorder; *GP* general practitioner; Angst, 2009: Comparison group Major Depressive Episode. ^1^Univariate analysis, ^2^Multivariable logistic regression.

Considering course and comorbidity features, the majority of studies found CD to have a higher risk of recurrence, to exhibit anxiety disorders and somatic comorbidity more often, and to use outpatient and inpatient care more frequently (Table 2). Among the assessed comorbid physical cardiovascular risk factors (CVRF), one study documented self-reported hypertension to occur more commonly in people with CD [4]. Except for family history of depression and education level, the majority of assessed variables in these studies were covariates or consequences rather than potential predictors of the persistence of depressive episodes, such as personality features, coping style, adverse life events prior to the onset of the chronic episode or specific depression symptoms during this episode. Among the rare community studies that assessed these variables, the prospective Zurich Cohort study [1] found participants with CD to more commonly report disturbed memory, feelings of inferiority, hopelessness, fear of everyday tasks, fear of being alone, thoughts of dying and lower levels of mastery than those with NCD. Participants of this study also revealed lower self-esteem at age 41, which was also observed in the NESARC study [3] (Table 2). The latter study did not observe an association between CD and childhood adverse events, whereas in the Australian survey CD was associated with traumatic load, death of someone close at the first depressive episode and current psychological distress [2].

Previous studies had several limitations that could partially explain inconsistent findings. Although major depressive disorder (MDD) is well known to be a heterogeneous diagnostic entity [7], the associations of specific depression symptoms and clinical subtypes of MDD with CD have not yet been established. Moreover, family history information was not collected using standardized instruments and the assessment of comorbid physical CVRF relied on self-reports. Accordingly, the present paper comparing people with CD, NCD and no mood disorder in the community aims to focus on specific atypical and melancholic depression symptoms and MDD subtypes, family history of mood disorders assessed through a standardized interview on all first-degree relatives, measured physical CVRF as well as personality traits, coping style, and adverse life-events in childhood and adulthood.

## Methods

### Participants

The data of the present paper stemmed from CoLaus|PsyCoLaus [8, 9], a prospective cohort study designed to study mental disorders and CVRF in the community and to determine their associations. The sample was randomly selected from the 35–75-year-old residents of the city of Lausanne (Switzerland) from 2003 to 2006 according to the civil register (participation 43%). Sixty-seven percent of the 35 to 66-year-old participants of the physical baseline exam (*n* = 5535) also accepted the psychiatric evaluation [9]. The gender distribution of the participants of the psychiatric exam did not differ significantly from that of the general population in the same age range [9]. Although the youngest 5-year band of the cohort was underrepresented and the oldest 5-year band overrepresented, participants and non-participants of the psychiatric exam had comparable scores on the General Health Questionnaire [10]. The analyses of the present paper were based on the baseline examination of the participants. Among the 3719 participants with physical and psychiatric evaluations at baseline, 101 participants were excluded: 94 with lifetime diagnoses of bipolar disorder (BPD), schizoaffective disorders, schizophrenia or schizophreniform disorder and 7 with incomplete data on MDD, resulting in a final sample of 3618 participants.

### Assessments

Diagnostic information on mental disorders was collected using the French version of the semi-structured Diagnostic Interview for Genetic Studies (DIGS)[11]. The DIGS was completed with the sections of the French version of the Schedule for Affective Disorders and Schizophrenia-Lifetime and Anxiety disorder version (SADS-LA) [12]. Additional questions were added to assess symptoms of atypical depression according to the DSM-IV specifier [13], which allowed us to subdivide MDD according to a history of atypical or melancholic features into four subtypes: 1) MDD with atypical features only, 2) MDD with melancholic features only, 3) combined MDD with atypical and melancholic features simultaneously or during distinct episodes, and 4) unspecified MDD with neither atypical nor melancholic features. For each depressive episode, information on timing, duration and treatment was elicited. Lifetime diagnoses were assigned according to the DSM-IV. CD was defined as MDD with a major depressive episode (MDE) that exceeded the duration of 24 months. The DIGS also collects information on socio-demographic characteristics, psychosocial functioning using the Global Assessment of Functioning (GAF) scale according to the DSM-IV. Family history information on BPD and MDD in all first-degree relatives was collected from participants using the Family History-Research Diagnostic Criteria (FH-RDC) [14]. The validity of the French version of the FH-RDC has been extensively tested [15]. Childhood adversity was assessed using the questions on exposure to traumatic events within the posttraumatic stress disorder (PTSD) section of the interview. Events during adulthood were elicited using the life-event interview of Amiel-Lebigre [16], which covers 52 potentially stressful life events as well as their timing and their negative affective impact ranging from 0 to 100, allowing us to compute cumulative severity scores [17].

Interviewers were master-level psychologists, who were trained over a one- to two-month period. Each interview and diagnostic assignment was reviewed by an experienced senior psychologist.

The personality dimensions of neuroticism and extraversion were evaluated using the Eysenck Personality Questionnaire [18], coping styles using the respective sections of the problem resolution strategy questionnaire [19], which were shown to measure emotion-focused coping, informal help-seeking behaviors (e.g., from partners, relatives, friends), and problem-focused coping for the French version [20].

Smoking status, physical activity (inactive if engagement in physical activity for less than 20 min twice a week), weight, height, waist circumference and blood pressure as well as venous blood samples to determine the levels of glucose, HDL-cholesterol, LDL-cholesterol and triglycerides were assessed at the physical investigation [8].

### Statistical analysis

Analyses were performed using the Statistical Analysis System (SAS) version 9.4 for Windows (SAS Institute, Inc., Cary, NC, USA). Multiple imputations were used for missing data (Makov Chain Monte Carlo method [21, 22] and Rubin’s multiple imputation strategy [23, 24]. Associations of sociodemographic, behavioral, course, symptom, comorbidity, healthcare use, family history, personality, and life-event variables with CD and NCD were established using multinomial logistic regression for analyses involving the three diagnostic groups with NCD as the reference. For analyses involving only participants with MDD, logistic regression was used. A first set of models (Model 1) was performed for each variable adjusting for age, sex, education and nationality. In a second step to establish the independent associations of each variable with CD and NCD, a multinomial logistic regression model (Model 2) was applied including all variables that were assessed in the three diagnostic groups reaching the lenient *p* < 0.1 level of significance according to Model 1. Similarly for participants with MDD, a logistic regression model (Model 2) was applied to add course and symptom characteristics. Variables such as low income, disability pension, marital status, GAF scores and healthcare use that were likely to be the consequence of rather than a risk factor for CD were not entered into Model 2. In the case of strong associations between two variables (number of depressive symptoms and suicidal ideation, decrease and increase in appetite/weight), only one of the two variables was entered into Model 2.

## Results

### Prevalence of CD and NCD

A proportion of 15.6% of our sample met lifetime criteria for MDD with an MDE (CD) longer than two years, and 29.3% met lifetime criteria for MDD without a chronic episode (NCD). The 12 months prevalence estimates for CD and NCD were 6.3 and 7.6%, respectively. Among those with CD the chronic episode lasted between 2 and 3 years in 28.6%, between 3 and 5 years in 29.7%, between 5 and 10 years in 24.7% and more than 10 years in 17.0%.

### Distribution of variables assessed in the three diagnostic groups

The distribution of variables assessed in the three diagnostic groups is provided in Table 3. According to the multinomial logistic regression models adjusted for socio-demographic characteristics (Model 1), compared to participants with NCD those with CD were more likely to be older, to have a disability pension and low income, to have made suicidal attempts, to have lower GAF scores, to have higher comorbid dysthymia, any anxiety disorder, generalized anxiety disorder, panic disorder or PTSD, to have consulted with a health professional or taken any medication or antidepressants more frequently, to have higher scores on the neuroticism and emotion-focused coping scales, but lower scores on the extraversion and informal help-seeking behavior scales. They also reported a higher impact of life events in adulthood and revealed a larger waist circumference. Moreover, NCD and participants with no MDD significantly differed in the distribution of all variables excepting nationality, education, former smoking, physical inactivity, lifetime prevalence of dysthymia and panic disorder, family history of BPD, problem-focused coping, and cardio-metabolic risk factors.

**Table 3:** Distribution of variables assessed in the three diagnostic groups (*N* = 3618)

	Chronic MDD (*n* = 563)		Non-chronic MDD (*n* = 1060)	No MDD (*n* = 1995)	
	% or mean (sd)	CD vs. NCD OR^c^ (95CI)	(ref)	% or mean (sd)	No MDD vs. NCD OR^c^ (95CI)
Socio-demographic characteristics					
Age (yrs)	51.5 (8.8)	1.23*** (1.11, 1.37)	49.7 (8.6)	51.4 (8.8)	1.27*** (1.17, 1.37)
Sex (female)	68.6	1.22° (0.98, 1.51)	63.7	43.0	0.42*** (0.36, 0.49)
Swiss nationality	70.7	0.86 (0.68, 1.10)	72.5	69.4	0.86° (0.72, 1.03)
Low education (compulsory school only)	19.4	1.21 (0.92, 1.60)	15.6	17.2	1.13 (0.91, 1.40)
Disability pension	9.8	2.27*** (1.50, 3.45)	4.2	2.7	0.54** (0.35, 0.81)
Low income (< CHF50′000.-/yr)	33.1	1.59*** (1.25, 2.02)	22.3	18.2	0.76** (0.63, 0.93)
Married	46.9	0.82° (0.66, 1.01)	52.3	66.0	1.59*** (1.36, 1.86)
Behavioral characteristics					
Smoking status					
Current	32.5	1.08 (0.86, 1.34)	31.6	25.2	0.72*** (0.61, 0.85)
Former	28.4	0.81° (0.64, 1.01)	32.5	32.8	0.91 (0.77, 1.07)
Physical inactivity^a^	48.7	1.23° (1.00, 1.52)	43.4	44.3	0.97 (0.83, 1.14)
Course					
Suicide attempts	12.8	1.67** (1.19, 2.36)	7.8	2.2	0.28*** (0.19, 0.41)
GAF score lifetime	68.2 (10.6)	0.64*** (0.58, 0.71)	73.2 (9.6)	81.8 (9.0)	2.88*** (2.61, 3.19)
GAF score current	68.7 (17.4)	0.69*** (0.63, 0.75)	76.0 (15.3)	83.8 (10.3)	1.99*** (1.82, 2.18)
Lifetime prevalence of other psychiatric disorders					
Dysthymia	5.2	1.67* (1.00, 2.79)	3.1	3.6	1.33 (0.87, 2.04)
Any anxiety disorder^b^	28.3	1.41** (1.11, 1.79)	21.9	11.3	0.50*** (0.41, 0.62)
Generalized anxiety disorder	5.7	2.37** (1.38, 4.08)	2.3	1.0	0.41** (0.22, 0.75)
Panic disorder	4.8	1.81* (1.06, 3.11)	2.8	1.8	0.76 (0.46, 1.27)
Agoraphobia	5.0	0.97 (0.60, 1.56)	4.9	2.5	0.59* (0.40, 0.89)
Social phobia	18.8	1.27° (0.96, 1.66)	15.7	7.5	0.48*** (0.38, 0.61)
PTSD	9.7	1.90** (1.28, 2.83)	5.1	1.4	0.29*** (0.18, 0.46)
Obsessive–compulsive disorder	2.5	1.72 (0.83, 3.58)	1.5	0.5	0.37* (0.16, 0.82)
Alcohol abuse or dependence	12.0	1.15 (0.83, 1.60)	11.6	11.3	0.71** (0.55, 0.91)
Illicit substance abuse or dependence	6.1	0.87 (0.57, 1.32)	8.3	5.0	0.52*** (0.38, 0.71)
Healthcare use					
Consultation with aprofessional	82.2	1.76*** (1.36, 2.28)	72.4	26.2	0.15*** (0.12, 0.17)
Any medication, %	66.6	1.32* (1.06, 1.64)	58.8	16.7	0.15*** (0.12, 0.17)
Antidepressants	48.1	1.49*** (1.21, 1.84)	37.5	6.3	0.12*** (0.10, 0.15)
Antipsychotics	3.6	1.46 (0.80, 2.66)	2.4	0.4	0.13*** (0.05, 0.30)
Mood stabilizers	0.9	0.89 (0.30, 2.65)	0.9	0.2	0.15** (0.04, 0.57)
Sedatives	48.8	1.14 (0.93, 1.41)	44.1	13.1	0.19*** (0.16, 0.23)
Hospitalization	11.7	1.35° (0.96, 1.89)	8.7	2.4	0.24*** (0.16, 0.34)
Familial history of mood disorders					
Bipolar disorder	4.2	1.21 (0.70, 2.09)	3.7	2.2	0.64° (0.40, 1.02)
MDD	45.0	0.84 (0.68, 1.04)	49.7	32.3	0.53*** (0.45, 0.63)
Personality characteristics					
Neuroticism	13.0 (5.3)	1.50*** (1.33, 1.70)	11.0 (5.5)	7.3 (4.8)	0.49*** (0.44, 0.54)
Extraversion	9.8 (4.9)	0.77*** (0.68, 0.87)	11.0 (4.7)	11.6 (4.6)	1.13** (1.03, 1.23)
Coping					
Emotion-focused coping	10.2 (3.8)	1.33*** (1.18, 1.51)	9.3 (3.8)	7.6 (3.6)	0.70*** (0.63, 0.77)
Help-seeking behavior	2.9 (2.2)	0.77*** (0.67, 0.88)	3.5 (2.1)	3.0 (2.1)	0.90* (0.82, 0.98)
Problem-focused coping	7.8 (1.8)	1.06 (0.94, 1.20)	7.6 (1.8)	7.7 (1.8)	1.03 (0.94, 1.13)
Life-events					
Childhood traumatic events	11.1	1.20 (0.86, 1.69)	9.4	5.1	0.57*** (0.43, 0.77)
Number of any life-events	13.8 (5.9)	1.09° (0.98, 1.20)	13.2 (5.3)	11.8 (4.7)	0.73*** (0.67, 0.79)
Impact adulthood lifetime events	602.8 (449.2)	1.24*** (1.13, 1.35)	490.0 (335.6)	329.6 (257.4)	0.54*** (0.49, 0.59)
Cardio-metabolic risk factors					
Waist circumference (cm)	87.3 (14.0)	1.16* (1.04, 1.30)	85.7 (12.8)	89.0 (12.9)	1.00 (0.92, 1.10)
Overweight	47.2	1.14 (0.92, 1.41)	42.9	51.4	1.09 (0.93, 1.27)
Diabetes	4.8	0.92 (0.56, 1.51)	4.6	5.6	0.80 (0.56, 1.15)
Dyslipidemia	32.6	1.12 (0.89, 1.41)	29.1	33.9	0.92 (0.77, 1.09)
Hypertension	31.8	1.23° (0.97, 1.57)	25.4	31.7	1.05 (0.88, 1.26)

*MDD* major depressive disorder; *CD* chronic MDD, *NCD* non-chronic MDD, *yr* year; *PTSD* post-traumatic stress disorder; *sd* standard deviation, *OR* odds ratio, *95CI* 95% confidence interval, *ref* reference

***p < 0.001, **p < 0.01, *p < 0.05, °*p* < 0.1

^a^Physically active less than 20 min twice a week. ^b^Generalized anxiety disorder, social phobia, panic disorder, or agoraphobia. ^c^Each line corresponds to one multinomial logistic regression model adjusted for age, sex, education and nationality (Model 1)

### Distribution of depression characteristics

According to logistic regression models (Model 1, Table 4), compared to participants with NCD those with CD had an earlier age of onset of MDD, and a higher risk to be in an episode at the time of the evaluation. They also reported a higher number of symptoms and experienced agitation, worthlessness/excessive guilt, diminished ability to think and concentrate, suicidal ideation/attempts more frequently, but were less likely to endorse changes in appetite/weight. In addition, they reported the melancholia symptom of excessive guilt more frequently but a decrease in appetite/weight less frequently. Finally, they more commonly reported the atypical symptoms increase in appetite/weight and leaden paralysis.

**Table 4:** Distribution of course and symptom characteristics among participants with MDD (*n* = 1623)

	Chronic MDD (*n* = 563)		Non-chronic MDD (ref) *n* = 1060) % or mean (sd)
	% or mean (sd)	OR^a^ (95CI)	
Course			
Age of onset (yrs)	31.2 (13.2)	0.68*** (0.61, 0.76)	34.5 (12.2)
Current episode	31.6	3.81*** (2.92, 4.97)	10.8
Recurrent episode	46.7	1.23 (1.00, 1.51)	41.5
Duration longest episode (yrs)	5.9 (5.5)	–^b^	0.6 (0.5)
Symptom manifestation during the most severe episode			
Duration (yrs)	5.3 (5.3)	–^b^	0.6 (0.4)
Number of symptoms	7.0 (1.4)	1.61*** (1.25, 2.07)	6.7 (1.4)
DSM-IV criteria for major depressive episode			
Depressed mood	98.2	1.23 (0.58, 2.60)	97.7
Diminished interest or pleasure	90.6	0.92 (0.64, 1.31)	91.0
Decreased or increased appetite/weight loss or gain	56.3	0.78* (0.63, 0.96)	61.7
Sleep disturbance	82.1	1.09 (0.84, 1.42)	80.8
Difficult falling asleep at least one hour	58.1	1.14 (0.92, 1.41)	54.7
Cannot fall asleep again	57.1	1.12 (0.91, 1.39)	53.2
Psychomotor agitation or retardation	74.8	1.17 (0.92, 1.48)	71.8
Agitation	45.3	1.30* (1.05, 1.60)	39.5
Retardation	41.8	1.03 (0.83, 1.27)	40.8
Fatigue or loss of energy	87.7	0.93 (0.68, 1.27)	88.0
Worthlessness or excessive guilt	78.3	1.51*** (1.19, 1.93)	71.0
Worthlessness	62.4	1.63*** (1.32, 2.02)	51.6
Diminished ability to think or concentrate	79.0	1.38* (1.08, 1.77)	73.2
Recurrent thoughts of death, suicidal ideation or suicidal attempts	51.9	1.90*** (1.54, 2.34)	36.4
Symptoms of melancholic features specifier			
Lack of reactivity	37.0	1.04 (0.84, 1.29)	35.7
Depression regularly worse in the morning	24.0	1.25 (0.97, 1.60)	20.2
Early morning awaking	31.2	1.21 (0.97, 1.53)	26.8
Decrease in appetite or reported weight loss	39.4	0.59*** (0.47, 0.73)	52.0
Excessive guilt	60.5	1.43*** (1.16, 1.77)	51.9
Symptoms of atypical features			
Mood reactivity	62.5	0.94 (0.76, 1.17)	64.3
Increase in appetite or reported weight gain	19.9	1.66*** (1.25, 2.21)	12.8
Hypersomnia	22.4	1.08 (0.84, 1.38)	21.8
Leaden paralysis	25.9	1.29* (1.01, 1.66)	20.7
Interpersonal rejection sensitivity	68.2	1.22° (0.98, 1.53)	64.4
Subtypes			
Atypical	16.3	1.29 (0.97, 1.72)	13.4
Melancholic	28.1	0.99 (0.79, 1.25)	28.2
Combined	11.7	1.16 (0.84, 1.62)	9.9
Unspecified	43.9	0.84 (0.68, 1.03)	48.4

*MDD* major depressive disorder, yrs  years, sd standard deviation, *OR* odds ratio, *95CI*  95% confidence interval, ref reference

***p < 0.001, **p < 0.01, *p < 0.05, *p* < 0.1

^a^Each line corresponds to one logistic regression model adjusted for age, sex, education and nationality (Model 1). ^b^Model does not converge due to quasi-compete separation of data points

### Associations of covariates according to the fully adjusted models

For variables assessed in all three diagnostic groups, the fully adjusted multinomial logistic regression (Model 2, Table 5) revealed that participants with CD reported a family history of MDD less frequently, scored higher on neuroticism but lower on the extraversion and help-seeking behavior scales and indicated a higher impact of adult life-events than those with NCD. Compared to participants with NCD, those with no MDD reported current smoking less frequently, were more likely to report dysthymia, but less likely to report social phobia, PTSD, suicidal attempts and family history of MDD. They also scored lower on the neuroticism and help-seeking behavior scales and indicated a lower impact on adult life events.

**Table 5:** Associations of socio-demographic and clinical correlates with diagnostic status in the whole sample and participants with MDD only according to the fully adjusted models (Model 2)

	All participants *N* = 3618		Participants with MDD *n* = 1623
	Chronic vs non-chronic MDD	No MDD vs non-chronic MDD	Chronic vs non-chronic MDD
	OR^b^ (95CI)	OR^b^ (95CI)	OR^c^ (95CI)
Behavioral comorbidities			
Smoking status			
Current	0.94 (0.72, 1.23)	0.73** (0.59, 0.91)	1.02 (0.77, 1.36)
Former	0.80 (0.62, 1.05)	0.83 (0.68, 1.01)	0.85 (0.64, 1.13)
Physical inactivity^a^	1.03 (0.82, 1.29)	1.13 (0.95, 1.35)	0.99 (0.78, 1.26)
Lifetime prevalence of other psychiatric disorders			
Dysthymia	1.42 (0.83, 2.44)	1.91** (1.20, 3.03)	1.65 (0.92, 2.98)
Generalized anxiety disorder	1.64 (0.91, 2.94)	0.74 (0.38, 1.44)	1.82 (0.97, 3.40)
Panic disorder	1.80 (0.98, 3.29)	1.14 (0.63, 2.06)	1.86 (1.00, 3.48)
Agoraphobia	0.62 (0.36, 1.06)	0.90 (0.55, 1.45)	0.60 (0.34, 1.07)
Social phobia	0.95 (0.70, 1.28)	0.68** (0.52, 0.89)	0.86 (0.62, 1.18)
PTSD	1.45 (0.92, 2.29)	0.44** (0.26, 0.75)	1.39 (0.86, 2.26)
Obsessive–compulsive disorder	1.36 (0.62, 3.00)	0.62 (0.25, 1.50)	1.33 (0.57, 3.11)
Alcohol abuse or dependence	0.97 (0.67, 1.40)	1.27 (0.95, 1.70)	0.98 (0.67, 1.45)
Illicit substance abuse or dependence	0.72 (0.45, 1.15)	0.77 (0.54, 1.10)	0.68 (0.42, 1.12)
Course			
Suicide attempts	1.15 (0.78, 1.67)	0.44*** (0.29, 0.68)	–
Familial history of mood disorders			
Bipolar disorder	1.03 (0.58, 1.83)	0.82 (0.49, 1.37)	0.95 (0.51, 1.76)
MDD	0.76* (0.60, 0.95)	0.65*** (0.54, 0.78)	0.74* (0.58, 0.94)
Personality characteristics			
Neuroticism	1.27** (1.08, 1.49)	0.52*** (0.46, 0.60)	1.12 (0.95, 1.32)
Extraversion	0.85* (0.74, 0.98)	1.02 (0.92, 1.14)	0.87 (0.76, 1.00)
Coping			
Emotion-focused coping	1.11 (0.95, 1.31)	1.08 (0.95, 1.23)	1.15 (0.98, 1.36)
Help-seeking behavior	0.82** (0.72, 0.95)	0.82*** (0.74, 0.91)	0.81** (0.70, 0.94)
Life-events			
Childhood traumatic events	1.00 (0.69, 1.46)	0.75 (0.54, 1.05)	0.86 (0.57, 1.29)
Impact adulthood lifetime events	1.19*** (1.07, 1.31)	0.65*** (0.59, 0.72)	1.15* (1.03, 1.28)
Cardio-vascular risk factors			
Waist (cm)	1.09 (0.97, 1.24)	0.99 (0.89, 1.10)	1.07 (0.93, 1.22)
Hypertension	1.13 (0.87, 1.47)	1.05 (0.86, 1.30)	1.12 (0.84, 1.49)
Course			
Age of onset (yrs)			0.58*** (0.50, 0.67)
Current episode			3.83*** (2.83, 5.21)
Recurrent episode			0.56*** (0.43, 0.74)
Symptom manifestation during the most severe episode			
DSM-IV criteria for major depressive episode			
Psychomotor agitation			1.26 (1.00, 1.60)
Worthlessness			1.13 (0.87, 1.45)
Diminished ability to think or concentrate			1.20 (0.91, 1.58)
Recurrent thoughts of death, suicidal ideation or suicidal attempts			1.47** (1.15, 1.87)
Symptoms of melancholic features specifier			
Depression regularly worse in the morning			1.17 (0.88, 1.55)
Early morning awaking			1.07 (0.82, 1.39)
Excessive guilt			1.12 (0.88, 1.43)
Symptoms of atypical features			
Increase in appetite or reported weight gain			1.55** (1.11, 2.15)
Leaden paralysis			0.93 (0.70, 1.25)
Interpersonal rejection sensitivity			0.94 (0.73, 1.22)

*MDD*   major depressive disorder, *yrs* years, *PTSD*   post-traumatic stress disorder, *OR* odds ratio; *95CI*  95% confidence interval

***p < 0.001, **p < 0.01, *p < 0.05

^a^Physically active less than 20 min twice a week. ^b^One single multinomial logistic regression model fully adjusted for age, sex, education, nationality and variables listed in the table (Model 2). ^c^One single logistic regression model fully adjusted for age, sex, education, nationality and variables listed in the table (Model 2)

The fully adjusted logistic regression model including depression-specific variables among participants with MDD (Model 2, Table 5) confirmed the associations observed according to the previous multinomial regression model except for neuroticism and extraversion that shortly failed to reach the level of statistical significance. With respect to the depression-specific variables, participants with CD had an earlier age of onset, were more likely to be in a current episode, were less likely to have had more than one depressive episode, and reported suicidal ideation/attempts and increase in appetite more frequently than those with NCD. However, the fully adjusted model revealed the other symptoms associated with CD according to Model 1including psychomotor agitation, the feeling of worthlessness or excessive guilt, diminished ability to think or to concentrate and leaden paralysis were not independently associated with chronicity.

## Conclusions

Based on a random community sample including more than 500 participants with CD and 1000 with NCD, using semi-structured diagnostic interviews conducted by psychologists, structured interviews to elicit family history information, validated questionnaires to establish personality traits and coping styles and measured cardio-metabolic characteristics, our study extends findings of previous partially conflicting results of population-based research on CD. Our most salient findings were that compared to NCD, CD is not only associated with less favorable socio-demographic characteristics but also with an earlier age of onset, a higher likelihood of being in a current episode, a lower likelihood of a family history of MDD, an increase in appetite or weight during the most severe episode, a higher exposure to life-events in adulthood as well as higher scores on neuroticism and lower scores on extraversion and the help-seeking scale. In contrast, our data did not provide evidence for differences in education level, childhood trauma or comorbidity with cardio-metabolic risk factors between the two forms of MDD.

As reported previously and extensively discussed, we have established a high lifetime prevalence of MDD in our sample as compared to previous epidemiological research, which was likely to be attributable to the particular features of our sample (urban area) and the diagnostic instrument (semi-structured interview with a low threshold to enter the depression section) [6]. With 34.7% of participants with CD among all participants with MDD, this proportion is at the upper bound of the range observed in previous studies [1–4].

One major finding was the opposite tendency for appetite and weight changes between CD und NCD. The Zurich Cohort study, the only study that previously reported on the distribution of depressive symptoms in the community, also documented a tendency for increased weight gain in CD and weight loss in NCD, although these differences did not reach the level of statistical significance in this smaller sample. Increase in appetite or weight during depression could be either a predictor for or a consequence of long-lasting episodes, whereas the more commonly reported suicidal ideation/attempts in participants with CD could be a consequence of the long duration of chronic episodes. Psychomotor agitation, worthlessness and excessive guilt, as well as leaden paralysis were also associated with CD in the models adjusted for socio-demographic variables. However, these associations failed to reach the level of statistical significance in the fully adjusted model. Hence, although these symptoms were not independently associated with CD and other symptoms, depression course or personality/coping characteristics partially accounted for their associations with CD, their occurrence was still related to the presence of CD.

We also found participants with CD to score higher on the personality dimension neuroticism but lower on extraversion, although these associations shortly failed to reach the level of statistical significance in the fully adjusted model that also included depression-specific variables. Our cross-sectional approach did not allow us to determine the nature of the relationship between personality scores and depression characteristics that interfered with the fully adjusted model. It is possible that characteristics of depression may have influenced the completion of personality scales, whereas conversely, pre-existing personality traits may have shaped the course and the manifestation of depression symptoms. In the latter case, adjustment for depression characteristics would not have been warranted. Associations between neuroticism and CD have already been reported in earlier clinical studies [25, 26]. Higher neuroticism scores could not only indicate vulnerability to depression but also reflect current state or a scar following the offset of depression [27]. Regarding coping styles, we found CD to be associated with lower help-seeking behavior, which might prevent these people from seeking adequate care, although the fact that they had already benefited from more formal care compared to those with NCD might have compensated for this. Previously, the Zurich Cohort study established an association between CD and low mastery [1].

Our findings of higher exposure to adult life stressors in people with CD are consistent with data from the Australian community study [2]. These stressors could predispose them to chronic episodes or maintain them [26]. In contrast to adult life stressors, the frequency of reported childhood trauma did not differ between CD and NCD in our sample. This observation is in line with that of the NESARC study [3] but contrasts with clinical research that showed CD to be associated with childhood trauma but not with childhood life events [28].

Applying a structured interview approach, we found a family history of MDD to be reported by participants with CD less frequently than those with NCD in the fully adjusted model. This observation contrasts with previous findings of Rubio et al. [3], which, however, were not based on structured interviews, and suggests genetic heterogeneity between CD and NCD. Regarding cardio-metabolic risk factors, our measured data did not provide evidence for differences between CD and NCD.

Consistent with previous community studies [1–4], we also observed less favorable socio-demographic characteristics such as lower income, a higher likelihood of receiving a disability pension in people with CD as compared to those with NCD, which are likely to be a consequence of a long-lasting depressive episode. However, similarly to the Zurich and the Australian study, we found the risk of CD to be independent of education level, whereas the findings of the NESARC and the Canadian studies suggested an association between lower educational level and CD. These discrepant findings could be due to country-specific factors. Our study also confirmed previous epidemiological findings of a higher risk of suicidal attempts, poorer psychosocial functioning, and a higher likelihood of comorbid anxiety but not substance use disorders in people with CD than those with NCD [1–4]. Similar to the Australian study [2], we established an earlier age of onset of MDD in participants with CD than in those with NCD, generally an indicator of more severe illness, although the Canadian study [4] and the NESARC [3] documented a later age of onset for CD. With 82.2% of people with CD reporting professional healthcare use in our study, this proportion was almost identical to the 81.8% in the Zurich Cohort study [1] and slightly higher than the 75.6% in the Canadian survey [4] and the 72.6% in the NESARC [3]. Our observation of a higher likelihood of professional healthcare use in people with CD than those with NCD is also consistent with previous findings from the community [1, 3] suggesting that lacking access to professional healthcare is an unlikely explanation for the persistence of episodes in these studies. More problematic is that only 47.8% of those with CD were treated with antidepressants. In the Zurich Cohort study, this proportion was only 30.9% and similarly low proportions were reported in studies that selected people with CD for treatment [29, 30].

The results of the present study need to be viewed in the context of several limitations. First, given the cross-sectional nature of our data, covariates that could be potential risk factors for chronic episodes may have been affected by the inaccurate recall or current or even remitted depressive episodes. Second, the restriction of the sample to the age range from 35 to 66 years reduced the generalizability of our findings. Third, the use of a sample from an urban area was likely to inflate our prevalence estimates [31].

Our findings have several clinical implications. Although the cross-sectional nature of our data did not allow us to determine temporal sequences, CD was associated with potentially modifiable risk factors such as personality features and coping strategies, which are accessible via psychotherapeutic approaches. Similarly, the deleterious effects of more frequent life stressors in patients with CD could be attenuated through psychotherapy. In addition, the observation that more than half of people with CD were not treated with antidepressants, although they generally consulted professional healthcare providers, is an intriguing finding. Timely prescriptions of antidepressants may have reduced the risk of chronic episodes or improved their course. In this respect, depressive episodes with agitation, increase in appetite, feelings of worthlessness and guilt, cognitive problems and suicidal ideation deserve particular clinical attention given that these symptoms are associated with chronicity.

## Data Availability

The data of CoLaus|PsyCoLaus study used in this article cannot be fully shared as they contain potentially sensitive personal information on participants. According to the Ethics Committee for Research of the Canton of Vaud, sharing these data would be a violation of the Swiss legislation with respect to privacy protection. However, coded individual-level data that do not allow researchers to identify participants are available upon request to researchers who meet the criteria for data sharing of the CoLaus|PsyCoLaus Datacenter (CHUV, Lausanne, Switzerland). Any researcher affiliated to a public or private research institution who complies with the CoLaus|PsyCoLaus standards can submit a research application to research.colaus@chuv.ch or research.psycolaus@chuv.ch. Proposals requiring baseline data only, will be evaluated by the baseline (local) Scientific Committee (SC) of the CoLaus and PsyCoLaus studies. Proposals requiring follow-up data will be evaluated by the follow-up (multicentric) SC of the CoLaus|PsyCoLaus cohort study. Detailed instructions for gaining access to the CoLaus|PsyCoLaus data used in this study are available at www.colaus-psycolaus.ch/professionals/how-to-collaborate/.
